# Comparison of Patient Outcomes Before and After Switching From Warfarin to a Direct Oral Anticoagulant Based on Time in Therapeutic Range Guideline Recommendations

**DOI:** 10.1001/jamanetworkopen.2022.22089

**Published:** 2022-07-14

**Authors:** Brian Haymart, Geoffrey D. Barnes, Xiaowen Kong, Mona Ali, Eva Kline-Rogers, Deborah DeCamillo, Scott Kaatz

**Affiliations:** 1 Division of Cardiovascular Medicine, Department of Internal Medicine, University of Michigan, Ann Arbor; 2 Department of Heart and Vascular Services, Beaumont Hospital, Royal Oak, Michigan; 3 Division of Hospital Medicine, Henry Ford Hospital, Detroit, Michigan

## Abstract

This cohort study evaluates stroke and major bleeding rates before and after switching from warfarin to a direct oral anticoagulant (DOAC) in patients grouped by pre-switch time-in-therapeutic range guideline thresholds.

## Introduction

Direct oral anticoagulants (DOACs), including apixaban, dabigatran, edoxaban, and rivaroxaban, are recommended instead of warfarin in patients newly diagnosed with nonvalvular atrial fibrillation (NVAF).^1,2^ However, it is less clear which established patients taking warfarin should switch to a DOAC. Medical societies have provided some guidance when considering switching to a DOAC based on time in therapeutic range (TTR): the European Society of Cardiology (TTR <70%), the American College of Chest Physicians (TTR <65%), and the American College of Cardiology (TTR <58%).^1,2,3^ Our objective was to use the Michigan Anticoagulation Quality Improvement Initiative clinical registry to evaluate stroke and major bleeding rates before and after switching from warfarin to a DOAC in patients grouped by pre-switch TTR guideline thresholds.

## Methods

The registry is part of a Blue Cross Blue Shield of Michigan–sponsored quality improvement collaborative.^4^ Funded abstractors at 6 participating anticoagulation clinics with institutional review board approval enter patient data into the registry. Our study cohort included patients initiated on warfarin for NVAF who were later switched to a DOAC between 2010 and 2021. Patients were excluded if NVAF was not the only indication for anticoagulation or if they did not have at least 6 months of follow-up after switching to a DOAC. The outcomes assessed were ischemic stroke and major bleeding based on International Society of Thrombosis and Hemostasis criteria.^5^ This report follows the STROBE reporting guideline. This study received a waiver of participant consent from the institutional review board because of minimal risk to participants.

TTR was calculated using the Rosendaal method,^6^ and patients were grouped based on guideline TTR thresholds. Each group’s outcome rates (number per 100 patient-years) while taking warfarin were compared with rates after switching to a DOAC. Rates of major bleeding were adjusted by a modified HAS-BLED (hypertension, abnormal renal/liver function, stroke, bleeding history or predisposition, labile INR [international normalized ratio], elderly, drugs/alcohol concomitantly) score (as continuous variable-removing the labile INR) while rates of ischemic stroke were adjusted by CHA_2_DS_2_-VASc score (as continuous variable) and compared using a mixed model with patients as random effect.^5^

Categorical variables were assessed using χ^2^ test or Fisher exact test. Two-sided *P* < .05 was considered significant for all comparisons. All statistical analyses were performed using SAS version 9.4 (SAS Institute) and R version 3.6.3 (R Project for Statistical Computing).

## Results

Of 6628 patients with NVAF taking warfarin, 524 (7.9%) meeting inclusion criteria switched to a DOAC. At the time of switch, the mean (SD) age was 73.3 (10.7) years, 231 (44.1%) were female, and 168 (35.2%) had at least moderate chronic kidney disease (creatinine clearance < 60 mL/min [to convert to mL/s/m^2^, multiply by 0.0167]) (Table 1). Apixaban was the predominant DOAC that patients switched to (277 patients [52.9%]), followed by rivaroxaban (146 patients [27.9%]).

**Table 1.** Patient Characteristics at Time of Switch From Warfarin to DOAC

Characteristic	Patients, No. (%) (N = 524)
Time receiving warfarin, median (IQR), y	1.28 (0.35-3.66)
Age at switch, mean (SD)	73.3 (10.7)
Age ≥65	411 (78.4)
Sex	
Male	293 (55.9)
Female	231 (44.1)
Weight, mean (SD), kg	88.7 (21.3)
BMI, mean (SD)	30.4 (6.4)
Underweight (<20)	12 (2.5)
Normal (20-25)	77 (15.7)
Overweight (>25)	401 (81.8)
Obese (30-40)	198 (40.4)
Severely obese (≥40)	34 (6.9)
HAS-BLED score, mean (SD)	3.1 (1.4)
0-2	168 (32.1)
≥3	356 (67.9)
CHA_2_DS_2_-VASc score, mean (SD)	4.0 (1.7)
0-3	190 (37.3)
≥4	320 (62.8)
TTR, % of time, mean (SD)	0.59 (0.22)
Comorbidities	
Prior major bleed	30 (5.7)
Hypertension	418 (79.8)
Chronic kidney disease	72 (13.7)
Serum creatinine	
Mean (SD), mg/dL	1.1 (0.8)
<1.5 mg/dL	413 (86.4)
Creatinine clearance, mean (SD), mL/min	78.5 (35.8)
<60	168 (35.2)
≥60	310 (64.9)
Liver disease	15 (2.9)
Prior ischemic stroke	9 (1.7)
Congestive heart failure	172 (32.8)
Diabetes	146 (27.9)
Peripheral artery disease	43 (8.2)
Myocardial infarction	87 (16.6)
Concomitant medications	
Antiplatelet(s)	261 (49.8)
PPI prescribed	176 (33.6)
CYP3A4/P-pg inducer	0
CYP3A4/P-pg inhibitor	134 (25.6)
DOAC prescribed	
Apixaban	277 (52.9)
Dabigatran	99 (18.9)
Edoxaban	2 (0.4)
Rivaroxaban	146 (27.9)
DOAC atrial fibrillation dosing	
Full	426 (81.3)
Reduced	80 (15.3)
Reason for switching	355(67.7)
Bleeding events	21 (5.9)
Clotting events	15 (4.2)
Unstable INRs	59 (16.6)
Adherence concerns with warfarin and/or INRs	25 (7.0)
Other clinical reasons	76 (21.4)
Patient preference	159 (44.8)

Abbreviations: BMI, body mass index; CHA_2_DS_2_-VASc, congestive heart failure, hypertension, age ≥75 years, diabetes, stroke or transient ischemic attack, vascular disease, age 65 to 74 years, sex category; DOAC, direct oral anticoagulants; HAS-BLED, hypertension, abnormal renal/liver function stroke, bleeding history or predisposition, labile INR, elderly, drugs/alcohol concomitantly; INR, international normalized ratio; TTR, time in therapeutic range.

SI conversion factors: To convert serum creatinine levels to micromoles per liter, multiply by 88.4; to convert creatinine clearance levels to mL/s/m^2^, multiply by 0.0167.

In all but the group with TTR less than 65%, the rate of major bleeding was significantly higher after switching (Table 2). The groups with TTR less than 70%, TTR less than 65%, and TTR less than 58% had major bleeding rates of 3.8, 4.9, and 5.8 per 100 patient-years while taking warfarin compared with 5.8, 6.3, and 7.5 per 100 patient-years after switching to a DOAC, respectively. Gastrointestinal hemorrhage accounted for 58.3% of bleeding before and 59.7% after switching, whereas 11.1% of bleeding before and 13.9% after switching were intracranial. In all TTR groups, there was no significant difference in ischemic stroke before and after switching. The group with TTR less than 70% had an ischemic stroke rate of 1.5 per 100 patient-years while taking warfarin and 2.0 per 100 patient-years after switching; for TTR less than 65%, the rates were 1.9 (before) and 1.6 (after); and for TTR less than 58%, they were 2.1 and 1.6.

**Table 2.** Event Rates Before and After Switching From Warfarin to a DOAC

	No. per 100 patient-years (95% CI)		*P* value
	Before switch (n = 524)	After switch (n = 524)	
**All switchers**			
Follow-up, median (IQR), d	467 (1208)	547 (1095)	NA
Major bleeds	3.0 (2.1-4.2)	5.8 (4.6-7.3)	<.001
Apixaban	3.4 (2.3-4.9)	3.8 (2.4-5.6)	.93
Dabigatran	3.0 (0.4-10.8)	4.4 (2.2-7.8)	.07
Rivaroxaban	1.9 (0.6-4.4)	10.3 (7.2-14.3)	<.001
Major bleed timing, after switch, months			
≤2	NA	4.6 (1.2-11.6)	NA
2-4	NA	8.0 (3.2-16.4)	NA
>4	NA	5.7 (4.4-7.4)	NA
Patients with major bleeds, No. (%)	31 (5.9)	64 (12.2)	<.001
Intracranial bleeds	0.34 (0.09-0.86)	0.81 (0.39-1.49)	.13
Ischemic strokes	1.0 (0.5-1.8)	1.8 (1.1-2.7)	.06
Ischemic stroke timing, after switch, months			
≤2	NA	4.6 (1.2-11.6)	NA
2-4	NA	3.4 (0.7-10.0)	NA
>4	NA	1.4 (0.8-2.3)	NA
Patients with ischemic strokes, No. (%)	12 (2.3)	21 (4.0)	.17
**Patients with TTR<70% while on warfarin (ESC guidelines)**			
No.	338	338	
Follow-up median (IQR), d	310 (1008)	548 (1095)	NA
Major bleeds	3.8 (2.4-5.7)	5.8 (4.2-7.7)	.03
Patients with major bleeds, No. (%)	20 (5.9)	40 (11.8)	.01
Ischemic strokes	1.5 (0.7-2.8)	2.0 (1.2-3.3)	.17
Patients with ischemic strokes, No. (%)	9 (2.7)	15 (4.4)	.30
**Patients with TTR<65% while on warfarin (ACCP guidelines)**			
No.	279	279	
Follow-up median (IQR), d	240 (746)	548 (1095)	NA
Major bleeds	4.9 (3.0-7.4)	6.3 (4.4-8.5)	.07
Patients with major bleeds, No. (%)	18 (6.5)	34 (12.2)	.03
Ischemic strokes	1.9 (0.8-3.6)	1.6 (0.8-2.9)	.74
Patients with ischemic strokes, No. (%)	8 (2.9)	9 (3.2)	1
**Patients with TTR<58% while on warfarin (ACC expert recommendation)**			
No.	210	210	
Follow-up median (IQR), d	176 (556)	548 (1277)	NA
Major bleeds	5.8 (3.2-9.7)	7.5 (5.3-10.4)	.01
Patients with major bleeds, No. (%)	11 (5.2)	32 (15.2)	.001
Ischemic strokes	2.1 (0.7-4.8)	1.6 (0.7-3.2)	.78
Patients with ischemic strokes, No. (%)	5 (2.4)	7 (3.3)	.77

Abbreviations: DOAC, direct oral anticoagulants; NA, not applicable; TTR, time in therapeutic range.

## Discussion

In this cohort study, patients had similar or higher rates of major bleeds and similar rates of ischemic stroke after switching to a DOAC, regardless of TTR threshold. This suggests that commonly cited TTR thresholds may not predict patients likely to benefit from a switch to DOAC therapy. The higher rates of bleeding on a DOAC is contrary to trial findings, possibly due to our patients being higher risk and having a different response to treatment. Limitations of this study include the potential for unmeasured confounding and attrition bias. Further research is needed to identify the best criteria for identifying NVAF candidates to switch from warfarin to a DOAC.
